# Beta IV spectrin inhibits the metastatic growth of melanoma by suppressing VEGFR2‐driven tumor angiogenesis

**DOI:** 10.1002/cam4.6522

**Published:** 2023-09-07

**Authors:** Eun‐A. Kwak, Tasmia Ahmed, Paola Cruz Flores, Hannah R. Ortiz, Paul R. Langlais, Karthikeyan Mythreye, Nam Y. Lee

**Affiliations:** ^1^ Department of Pharmacology University of Arizona Tucson Arizona USA; ^2^ Department of Chemistry & Biochemistry University of Arizona Tucson Arizona USA; ^3^ Department of Medicine University of Arizona Tucson Arizona USA; ^4^ Department of Pathology University of Alabama at Birmingham Birmingham Alabama USA; ^5^ Comprehensive Cancer Center University of Arizona Tucson Arizona USA

**Keywords:** angiogenesis, endothelial cells, tumor vascularization, vascular growth factor, β_IV_‐spectrin

## Abstract

**Background:**

Tumor‐associated angiogenesis mediates the growth and metastasis of most solid cancers. Targeted therapies of the VEGF pathways can effectively block these processes but often fail to provide lasting benefits due to acquired resistance and complications.

**Results:**

Recently, we discovered β_IV_‐spectrin as a powerful regulator of angiogenesis and potential new target. We previously reported that β_IV_‐spectrin is dynamically expressed in endothelial cells (EC) to induce VEGFR2 protein turnover during development. Here, we explored how β_IV_‐spectrin influences the tumor vasculature using the murine B16 melanoma model and determined that loss of EC‐specific β_IV_‐spectrin dramatically promotes tumor growth and metastasis. Intraperitoneally injected B16 cells formed larger tumors with increased tumor vessel density and greater propensity for metastatic spread particularly to the chest cavity and lung compared to control mice. These results support β_IV_‐spectrin as a key regulator of tumor angiogenesis and a viable vascular target in cancer.

## 1 INTRODUCTION

Antiangiogenic therapies are considered an integral part of standard care for many cancer treatments as they can either block the formation of new tumor vessels or effectively normalize them to improve tumor perfusion and drug delivery.^1^, ^2^, ^3^, ^4^, ^5^ Drugs like bevacizumab and sorafenib are able to slow tumor progression by interfering with the vascular endothelial growth factor (VEGF) and its receptor, VEGFR2, although these and other anti‐VEGF therapies have often shown limited efficacy in long‐term treatments due to acquired resistance and toxicities.^6^, ^7^, ^8^, ^9^ Studies have shown that VEGF‐based therapies may be susceptible to such limitations given that VEGFR2 can sometimes become activated independent of VEGF ligands, thereby bypassing the effects of bevacizumab, or in the case of kinase inhibitors, yield off‐target effects.^7^, ^10^, ^11^, ^12^ Hence, defining new VEGF‐dependent targets will be a crucial factor in developing more effective antiangiogenic therapies.

We recently discovered an entirely new regulatory mechanism of VEGF/VEGFR2 signaling based on a novel cytoskeletal signaling complex.^13^, ^14^ We reported that β_IV_‐spectrin, a large membrane scaffold protein previously characterized mostly in the nervous system and heart, is dynamically expressed in vascular endothelial cells (EC) to regulate sprouting angiogenesis during development.^13^, ^14^ Early loss of endothelial‐specific β_IV_‐spectrin in mice resulted in enhanced embryonic lethality due to hypervascularization and hemorrhagic defects whereas depletion at neonatal stages produces greater vessel density and tip cell populations in developing retina. Notably, we observed that β_IV_‐spectrin expresses primarily in stalk cells to inhibit their tip cell potential by enhancing VEGFR2 turnover through recruitment of CaMKII to the plasma membrane, which directly phosphorylates VEGFR2 at Ser984, a previously undefined phosphoregulatory site that strongly induces VEGFR2 internalization and degradation.^13^

While the above findings established critical new facets of VEGF signaling regulation, they also raised important questions regarding the role of β_IV_‐spectrin in pathologic contexts, including tumor‐associated angiogenesis. Here we address how β_IV_‐spectrin influences tumor growth and progression by controlling the vasculature.

## 2 MATERIALS AND METHODS

### 2.1 Generation of EC‐specific inducible β_IV_ ‐spectrin knockout mice

β_IV_‐spectrin Fl/Fl mice were derived from C57/Bl6 and were generous gifts from Dr. Thomas Hund (Ohio State University).^15^, ^16^ EC‐specific Cre mouse model (Cdh5(PAC)‐CreERT2) was purchased from Taconic. To generate and induce β_IV_‐spectrin knockout in β_IV_‐EC^KO^ mice, β_IV_‐spectrin Fl/Fl and Cdh5(PAC)‐CreERT2 lines were crossed and genotyped as described.^13^ All experiments were performed using both male and female mice between 14 and 18 weeks of age. All animal procedures were performed in accordance with the guidelines approved by the University of Arizona Institutional Animal Care and Use Committee (Protocol # 2021‐0772).

### 2.2 Tamoxifen‐induced EC‐specific β_IV_ ‐spectrin knockout and tumor injection

Genotyped male and female control (Cdh5(PAC)‐CreERT2) and β_IV_‐EC^KO^ (Fl/Fl‐Cdh5(PAC)‐CreERT2) mice were injected with 100 mL of tamoxifen (5 mg/mL) by IP at both 10 and 5 days prior to tumor cell injection. At Day 0, each mouse group were injected via IP with mycoplasma‐tested B16‐F0 cells (1 × 10^5^ cells per mouse). Mice were monitored for palpable tumor growth at indicated time points and tumor volumes estimated using calipers and the standard formula [(width)^2^ × length]/2.

### 2.3 Isolation of primary ECs from B16‐F0 tumors

Primary ECs were isolated from B16‐F0 tumors grown in control and β_IV_‐EC^KO^ mice. At Day 17 of tumor growth studies, mice were sacrificed, and the resected tumor tissues were finely chopped and then transferred to collagenase solution (1 mg/mL collagenase in 1× DMEM with 0.2 μm syringe filter) for enzymatic digestion for 45 min at 37°C. Digested tissues were passed through a 15 g needle 10× before being strained through a 70 mm cell strainer. Strained and washed tissue solutions were centrifuged at 4°C at 1000 rpm for 5 min. The pellet was resuspended in 0.1% BSA in PBS and incubated with CD31 antibody‐conjugated Dynabeads for 15 min at room temperature with gentle shaking then washed with 0.1% BSA/PBS 5×. The beads were resuspended in 1 mL of EC Media [MCDB‐131 Medium (Gibco) with 10% (v/v) fetal bovine serum, 2 mM l‐glutamine (Gibco), 1 mM sodium pyruvate (Gibco), 100μg/mL of heparin (Sigma‐Aldrich), and endothelial cell growth supplement (Sigma‐Aldrich)] with 1× penicillin streptomycin and plated on gelatin coated cell culture flasks. Upon 2 days, cells were separated from beads with trypsin (Gibco).

### 2.4 Western blotting

Cell lysates were separated by SDS‐PAGE and electrophoretic transferred onto the PVDF (polyvinylidene difluoride) membranes (BIO‐RAD). Transferred membranes were blocked with 5% skim milk in TBS with 0.1% Tween‐20, and then incubated with primary antibodies at 4°C overnight. Following day, membranes were washed three times in TBS buffer with 0.1% Tween‐20 and incubated with the secondary antibody for 45 min at room temperature. Membranes were washed five times in TBS buffer with 0.1% Tween‐20 each 5 min then imaging by ChemiDoc Imaging system (BIO‐RAD). The following antibodies were purchased from Cell Signaling Technology [(VEGFR2‐ #9698, p‐AKT S473‐ #4060, p‐p44/42‐ #9101, p‐p38‐ #3871, p‐PLCγ2‐#4511, neuropilin 1‐ #3725)]. Antibodies to β_IV_‐spectrin and β‐actin were purchased from Santa Cruz Biotechnology (#SC514744) and Sigma‐Aldrich (#A1978) respectively. VEGFR1 antibody was purchased from R&D Systems (#AF471).

### 2.5 Immunohistochemical staining and analysis of B16‐F0 tumors

Immunohistochemical (IHC) detection of EC‐antigen (CD31) in formalin‐fixed, paraffin‐embedded B16‐F0 tumor sections (5 μm) was performed by de‐paraffinization and antigen retrieval (heated in citrate buffer pH 6). Samples were incubated with CD31 (Abcam #Ab7388) at 4°C overnight. Then samples were incubated with peroxidase‐coupled secondary for 30 min at room temperature and developed with AEC solution for 10 min. Nuclei were stained with hematoxylin for 1 min. Samples were imaged with a Nikon SMZ800N microscope, then analyzed using ImageJ and manual quantification of CD31‐positive vessels in at least three visual microscopic fields per tumor sample (per mouse).

### 2.6 Statistics and reproducibility

Statistical analysis was performed using an unpaired two‐tailed Student's *t*‐test. Significant statistical differences between groups were indicated as: *p < 0.05. Data are presented as mean ± SEM. No statistical method was used to predetermine sample size. Statistical analyses and graphics were carried out with GraphPad Prism software and Microsoft Excel.

## 3 RESULTS

### 3.1 EC‐specific β_IV_ ‐spectrin deletion enhances B16 tumor growth

**FIGURE 1:**
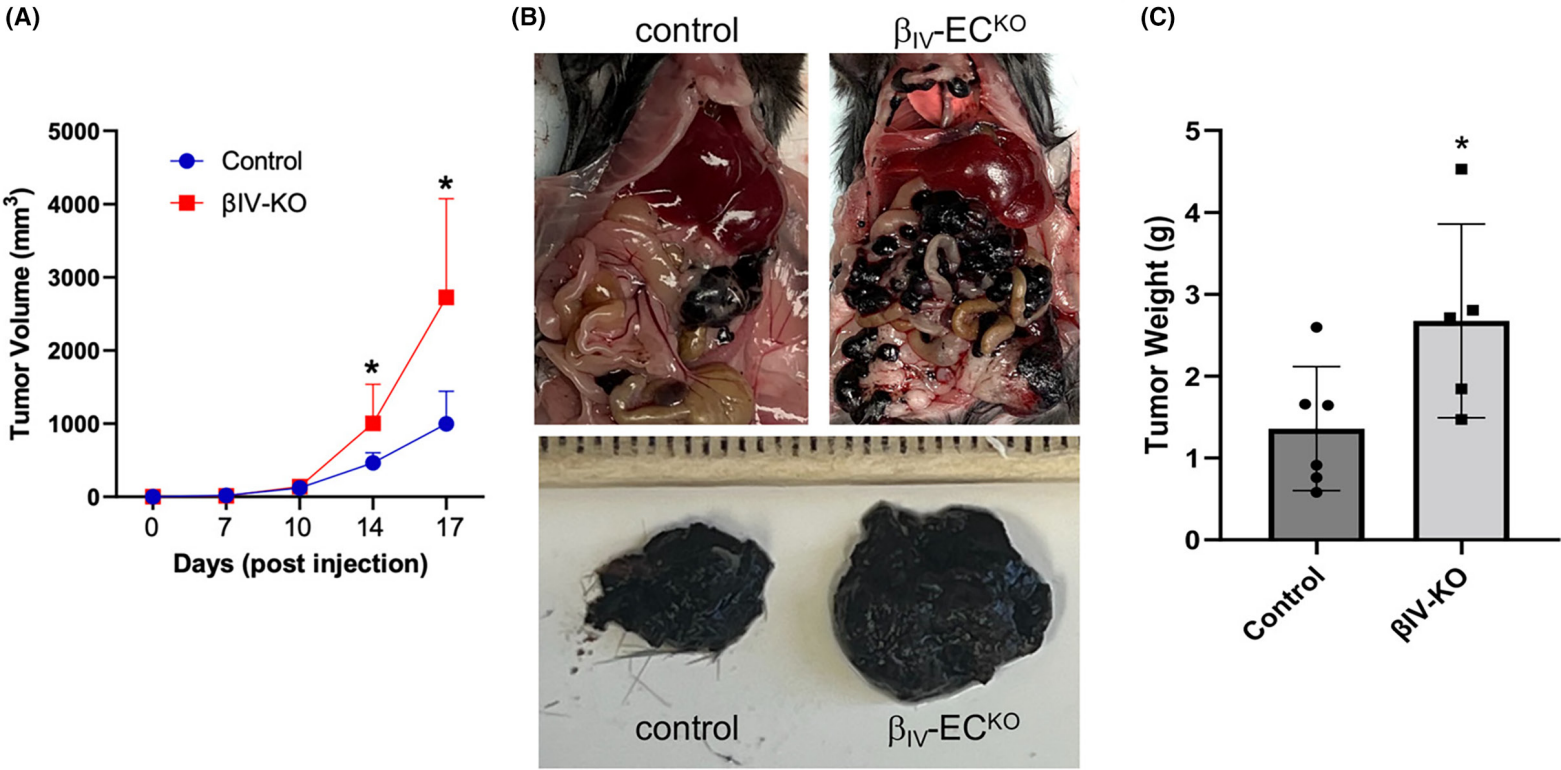
Loss of EC‐specific β_IV_‐spectrin expression promotes B16‐F0 tumor growth. (A) Graph represents the average tumor volume measured by calipers at indicated days post‐injection. Data based on *n* = 8 (control) and *n* = 7 (β_IV_‐EC^KO^) mice with error bars represented in SEM.*p < 0.01 relative to control groups. (B) Representative images of B16‐F0 tumor bearing control and β_IV_‐EC^KO^ mice at necropsy (upper panels) and zoomed‐in images of isolated individual tumor nodules (lower panels). (C) Graph shows average tumor weight of consolidated tumors per mouse between the two groups. *p = 0.026 relative to control mice.

To investigate the role of β_IV_‐spectrin in tumor progression, we chose the murine B16 melanoma cells for their aggressive growth and metastatic properties.^17^ Given that intravenous injection of B16‐F0 cells preferentially accumulate in the lung and liver,^18^ we instead performed intraperitoneal (IP) injections on an EC‐specific tamoxifen‐inducible β_IV_‐spectrin knockout mouse strain we generated.^13^, ^14^ Using this mouse model, we previously showed that β_IV_‐spectrin is essential for sprouting angiogenesis during embryonic development and postnatal retinal vascularization but otherwise is dispensable in mature quiescent vessels of adult mice. Indeed, tamoxifen‐induced β_IV_‐spectrin gene deletion in adult mouse vasculature does not promote angiogenesis, vascular leakage, or remodeling, thus rendering this model suitable for studies on tumor‐associated angiogenesis. In the present study, the Cre recombinase was first induced by administering tamoxifen via IP injection in control (Cdh5‐ERT2) and β_IV_‐spectrin knockout (β_IV_‐EC^KO^; Fl/Fl‐Cdh5‐ERT2) mice prior to inoculation with B16‐F0 cells. Upon injection, both mouse groups formed similarly palpable tumors within 10 days although by Day 14 the β_IV_‐EC^KO^ mice displayed discernably larger tumors (Figure 1A). Necropsy following the endpoint of the study (Day 17) showed a striking increase in tumor growth throughout the abdominal cavity in β_IV_‐EC^KO^ compared to control mice (Figure 1B,C).

### 3.2 Loss of EC‐specific β_IV_ ‐spectrin promotes tumor vascularization

**FIGURE 2:**
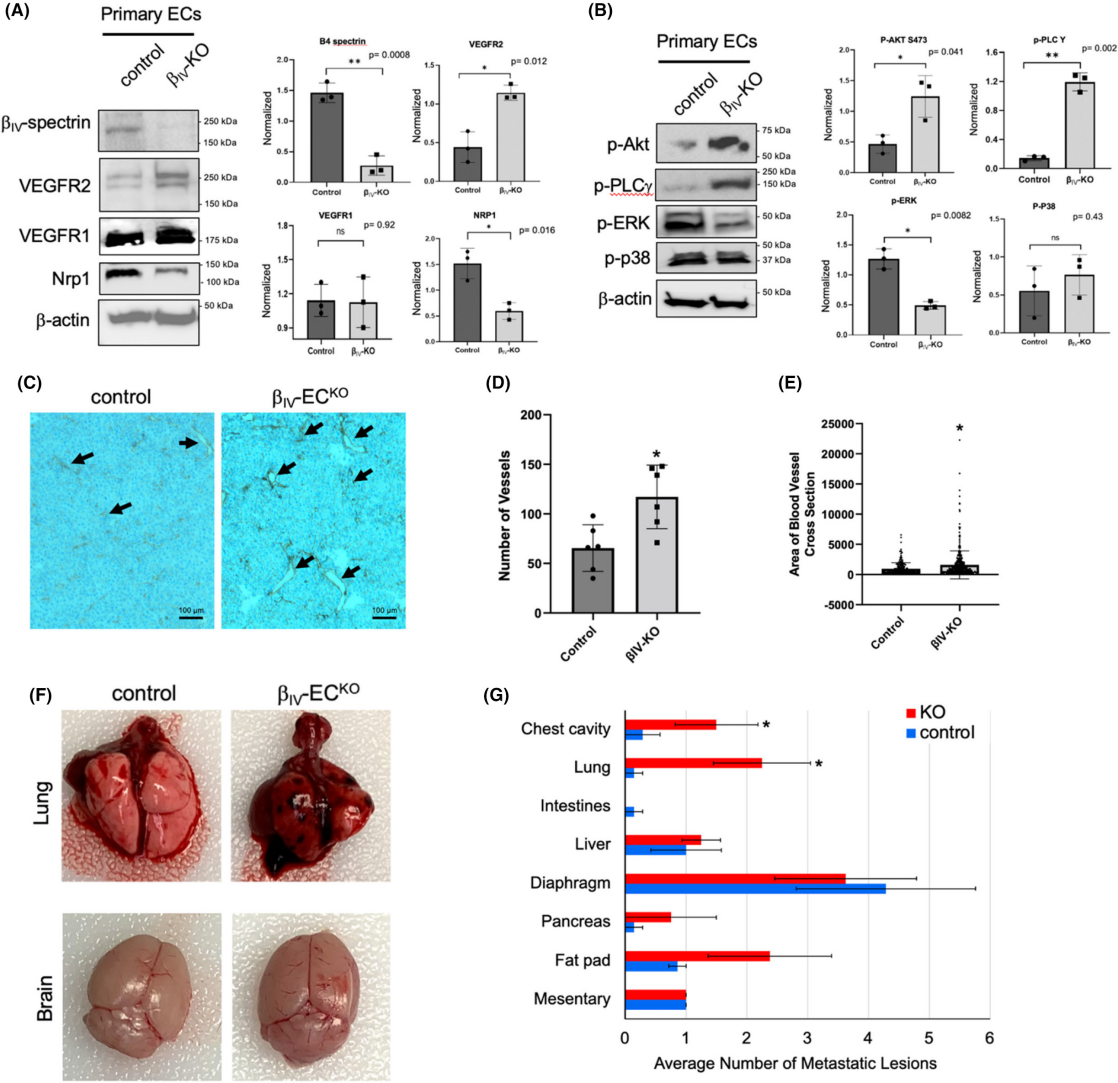
Tumor vascularization and metastasis are enhanced in β_IV_‐EC^KO^ mice. (A, B) Immunoblots show the level of endogenous β_IV_‐spectrin along with the indicated receptor levels and activation of VEGF signaling effectors in freshly isolated primary ECs from control versus β_IV_‐EC^KO^ tumors. Graphs represent densitometry quantifications based on three biological repeats normalized to β‐actin loading control. *p* values are indicated in each graph. (C) Shown are representative IHC images of tumor sections stained with CD31 (indicated by black arrows). (D, E) Graphs show the number of vessels counted per mouse tumor section and the cross‐section area of individual blood vessels (*n* = 6 mice for control and β_IV_‐EC^KO^). * *p* = 0.003 relative to control group. (F) Shown are representative images of lung and brain isolated from control and β_IV_‐EC^KO^ mice at necropsy. (G) Graph represents the average number of metastatic tumor nodules on the surface of different tissues per mouse (*n* = 7 mice for control and *n* = 8 for β_IV_‐EC^KO^ mice). Error bars represented by SEM. *p < 0.04 relative to control.

Since we previously observed that loss of EC‐specific β_IV_‐spectrin expression results in VEGFR2 upregulation and hypervascularization during normal development,^13^ here we assessed whether the enhanced tumor progression in β_IV_‐EC^KO^ mice was primarily attributed to increased angiogenesis. To do so, we first isolated primary ECs from the tumors of control and β_IV_‐EC^KO^ mice to gauge the efficiency of β_IV_‐spectrin deletion as well as for β_IV_‐spectrin‐dependent changes in VEGFR2 levels. As observed previously in developing mouse retinal vasculature, there was a marked increase in VEGFR2 but not VEGFR1 protein levels upon loss of β_IV_‐spectrin expression in the freshly isolated tumor ECs compared to control (Figure 2A). Interestingly, neuropilin 1 (Nrp1), a coreceptor of VEGFR2 signaling, was downregulated upon loss of β_IV_‐spectrin (Figure 2A; fourth panel) although this had no significant impact on downstream signaling as shown by the increased activation of Akt and PLCγ (Figure 2B), which represent some of the key VEGFR2 pathways to become activated during angiogenesis. But not all canonical VEGFR2 pathways showed increased activation in β_IV_‐EC^KO^ tumor ECs including ERK and p38 (Figure 2B), likely due to the reduced VEGFR2 receptor internalization upon loss of β_IV_‐spectrin, which can dampen MAPK signaling as we previously demonstrated.^13^, ^14^

In parallel studies, immunohistochemical (IHC) analysis using hematoxylin staining and an EC marker, CD31, showed a significant increase in the overall microvascular density in β_IV_‐EC^KO^ mouse tumors (Figure 2C; black arrows). Quantification of these tumor sections revealed a nearly twofold increase in the average number and diameter of these vessels in β_IV_‐EC^KO^ tumors relative to control (Figure 2D,E). Taken together, the biochemical and IHC data strongly suggested a key role of β_IV_‐spectrin in regulating the tumor vasculature in terms of overall vessel density and size.

### 3.3 β_IV_ ‐spectrin suppresses B16 tumor metastasis to the lung and chest cavity

Next, to determine the effects of β_IV_‐spectrin regulation of tumor angiogenesis on metastasis, we assessed for the presence of visible tumor nodules on the surface of the major organs and tissues. While necropsy showed similar levels of metastatic spread from the primary tumors to the liver, diaphragm and the mesentery, the number of metastatic lesions found on the surface of the lung and chest cavity were greatly elevated upon loss of β_IV_‐spectrin (Figure 2F; upper panels). But despite brain metastasis also being observed frequently in melanoma, there were no overt signs of metastatic growth in control or β_IV_‐EC^KO^ brains (Figure 2F; lower panels). Instead, we observed increased metastatic spread in β_IV_‐EC^KO^ mice to the pancreas and fat pad, albeit to a lesser degree than the lung (Figure 2G). Taken together, the data here supported the role of β_IV_‐spectrin in suppressing tumor growth and metastasis through inhibition of angiogenesis.

## 4 DISCUSSION

Our recent discovery of β_IV_‐spectrin as an EC marker critical for normal vascular development prompted us to investigate how this specialized cytoskeletal protein affects tumor progression. Our work here marks the first study of β_IV_‐spectrin‐dependent effects in any kind of cancer and the results clearly demonstrate its role in suppressing tumor vascularization, growth and metastasis.

Given that β_IV_‐spectrin acts as an endogenous suppressor of vascular sprouting during development, it is not surprising that tumor angiogenesis is also inhibited. However, this was not a forgone conclusion since, at least in developing retinal vasculature, β_IV_‐spectrin expression is mostly confined to a narrow band of stalk cells, but not tip cells, near the radial front of vascular sprouting.^13^ Thus, precisely how this intricate spatiotemporal expression observed during retinal development would compare to the much more chaotic and complex tumor microenvironment was less than predictable. While we also attempted to visualize β_IV_‐spectrin expression in the B16 tumor vessels, our IHC staining proved unsuccessful, potentially due to the low abundance of this protein and its highly dynamic expression pattern. However, the fact that the β_IV_‐spectrin expression was confirmed in tumor‐isolated primary ECs generally support that the loss of this protein enhances tumor progression through neovascularization.

It is interesting to note that the metastatic properties between the two groups were similar except to the chest cavity and lung. Indeed, while it is well documented that B16 tumors have a propensity to metastasize to the lung,^18^ it is unclear whether this preferential lung metastasis in β_IV_‐EC^KO^ compared to control mice is due to greater tumor cell dissemination or the accelerated tumor growth at these metastatic sites. But given that the fat pad and pancreas were some of the other regions also affected by β_IV_‐spectrin, it is reasonable to conclude that this protein inhibits the metastatic spread of B16 tumor cells to many but not all regions.

While many other angiogenic targets besides VEGF and VEGFR2 are currently under clinical evaluations, the VEGF/VEGFR2 pathway still represents one of the most important vascular targets and thus further improvements on existing VEGF‐based therapies are likely to provide more rapid advances in standard care for many cancer patients. Our findings here suggest that stimulating β_IV_‐spectrin expression in the vascular system could be a viable approach to suppress endogenous VEGF signaling as an alternative to targeting VEGF‐A or the VEGFR2 kinase.

## AUTHOR CONTRIBUTIONS

**Eun‐A. Kwak:** Data curation (lead); formal analysis (lead); investigation (lead); methodology (lead); writing – original draft (equal). **Tasmia Ahmed:** Data curation (supporting); methodology (supporting). **Paola Cruz Flores:** Data curation (supporting); formal analysis (supporting); investigation (supporting). **Hannah R. Ortiz:** Data curation (supporting); formal analysis (supporting). **Paul R. Langlais:** Data curation (supporting); writing – review and editing (supporting). **Karthikeyan Mythreye:** Conceptualization (supporting); formal analysis (supporting); writing – original draft (supporting); writing – review and editing (equal). **Nam Y. Lee:** Conceptualization (lead); formal analysis (supporting); project administration (lead); supervision (lead); writing – original draft (equal); writing – review and editing (equal).

## CONFLICT OF INTEREST STATEMENT

The authors declare no conflicts of interest.

## Data Availability

Not applicable.
